# Exploring the Impact of Lipid Structure and Composition on the Digestion of Next-Generation Meat and Dairy Analogues

**DOI:** 10.3390/foods15040772

**Published:** 2026-02-20

**Authors:** Zarnab Asif, Clive A. Prestidge, Paul Joyce

**Affiliations:** 1 Centre for Pharmaceutical Innovation, School of Pharmacy & Biomedical Sciences, College of Health, Adelaide University, Adelaide, SA 5000, Australia; zarnab.asif@adelaide.edu.au (Z.A.); clive.prestidge@adelaide.edu.au (C.A.P.); 2 ARC Industry Transformation Training Centre (ITTC) for Facilitated Advancement of Australia’s Bioactives (FAAB), School of Natural Sciences, Macquarie University, Sydney, NSW 2109, Australia

**Keywords:** plant-based, meat analogues, plant proteins, sustainable foods, lipolysis, fatty acids, fat analogues

## Abstract

The world population is increasing exponentially and is expected to reach 9.2 billion people by 2040, intensifying pressures on food systems and raising concerns regarding food security and environmental sustainability. In response, plant-based and microbially sourced meat and dairy analogues have emerged as alternatives to animal-derived foods. These next-generation products rely heavily on fat substitutes to replicate the sensory and functional roles of animal fats, which not only influence flavour, texture, and consumer acceptance but also play a critical role in digestion and the absorption of lipophilic nutrients. This review advances a structure–interface–digestion framework for understanding fat substitutes in meat and dairy analogues, in which lipid composition and supramolecular organization jointly determine digestive fate and nutritional functionality. Rather than acting solely as sensory replacers, fat analogues regulate lipolysis kinetics, mixed micelle formation, and the bioaccessibility of lipophilic nutrients through key parameters including fatty acid chain length, degree of saturation, physical state, and interfacial architecture. Within this framework, plant and microbially derived lipid systems are not functionally interchangeable with animal fats and therefore require purposeful structural design to ensure effective digestion and nutrient delivery. By integrating insights from food sciences, nutrition, and biotechnology, this review highlights the necessity of rationally engineered fat analogue systems that reconcile sustainability constraints with sensory performance and optimal nutritional efficacy.

## 1. Introduction

The global population is increasing rapidly, triggering significant food insecurity concerns [1]. Almost a billion people in the world are unable to afford food with sufficient protein and calorie levels required for a healthy lifestyle. Due to the deficiency of these essential nutrients, serious health consequences are prevalent, such as muscle weakness, defective immune system, and deficiency in growth [2]. Furthermore, increased consumption of meat products is leading to unsustainable greenhouse gas emissions that are rapidly contributing to climate change [3]. To overcome these challenges and to feed the global population in an environmentally friendly manner, alternative meats and dairy have been developed to serve as a nutritional alternative to animal proteins and fats [4]. However, while considerable research has focused on protein digestion and absorption in these meat/dairy analogues, research on fat digestion and absorption remains limited, despite the fact that fats play a significant role in the nutritional quality, sensory characteristics, and health impacts of these alternative foods [5].

In particular, the type of fat present in meat/dairy analogues is integral to the mouthfeel and overall sensory experience, further emphasizing the need for a deeper understanding of fat digestion in plant-sourced foods [6]. Furthermore, fats present in meat/dairy analogues contribute to the overall nutritional profile, providing essential fatty acids, vitamins, and fat-soluble nutrients. Subsequently, the nutritional profile of the various meats or dairy products is significantly impacted by the type of lipids present. Traditional animal fats, such as those in meat and dairy, have highly specific fatty acid profiles and supramolecular structures, such as the milk fat globule membrane, which confer distinct digestive behaviours and nutritional outcomes [7]. Replicating these complex characteristics in analogue fats is a major scientific and industrial challenge.

Plant oils and microbial-derived fats are the dominant sources of lipid ingredients for current analogues, yet they diverge significantly from animal fats in both composition and structure. Plant-derived oils are generally higher in polyunsaturated fatty acids (PUFAs) and lack the crystallization and structural organization typical of animal fats, (some plant-sourced lipids like coconut and palm have high proportions of saturated fatty acids, ranging from 48 to 90% and are in crystalline form at ambient or refrigeration temperature, that is why they are frequently used in alternative meats and dairy products for providing functional attributes similar to real animal fats), while microbial-derived lipids produced via precision fermentation offer new opportunities for tailoring fatty acid composition but remain underexplored in terms of digestive fate. As such, their impact on lipolysis, micelle assembly, and nutrient bioaccessibility remains poorly characterized [8].

Various experimental models, such as advanced in vitro lipolysis assays, are used to measure the lipid digestion and absorption of these meat and dairy analogues [9]. During lipolysis, triglycerides breakdown into free fatty acids and monoglycerides in the digestive tract, which together with endogenous phospholipids and bile salts form mixed micelles. The size and composition of these micelles largely affects the efficiency of lipid absorption [10,11]. Meat and dairy analogues containing structured fats or emulsified lipid systems exhibit distinct digestion kinetics compared with unstructured oils. These differences influence the rate and extent of lipid breakdown, the formation of mixed micelles, and, ultimately, the absorption of lipophilic nutrients. In meat and dairy analogues, combinations of saturated and unsaturated plant oils can give rise to distinct lipid architectures, such as partial fat encapsulation, which may facilitate enzymatic access and digestion. However, the synergistic effects of mixed lipid systems remain insufficiently understood, particularly in complex food matrices where protein–lipid interactions can further modulate lipid emulsification, interfacial composition, and lipase accessibility, thereby altering lipid digestion behaviour [12,13,14].

Given that many vitamins and micronutrients are lipid soluble, the type and structure of fats present (e.g., saturated/unsaturated and emulsified/non-emulsified) is subsequently crucial for their efficient absorption. This is because fat-soluble compounds rely on the formation of mixed micelles and colloidal vesicles that result from the digestion of triglycerides into fatty acids and monoglycerides to facilitate their solubilization and absorption across the small intestine. If sufficient fat is not present, the levels of mixed micelles present in the small intestine are too low to facilitate solubilization, which ultimately reduces lipid-soluble vitamins and micronutrient bioavailability [15]. Since this type of nutrient absorption is strongly dependent on lipid digestion, it is essential to critically examine how analogue fats behave within the gastrointestinal tract. Differences in chain length, saturation, droplet size, and interfacial composition may all influence the rate and extent of lipolysis and, consequently, the efficiency of nutraceutical absorption. A deeper understanding of these processes will allow researchers and industry to design next-generation fat analogues that provide not only sensory and functional equivalence to animal fats but also nutritional adequacy and health benefits. This review addresses these questions by examining current knowledge on the digestion of meat and dairy fat analogues, evaluating methodological approaches, and identifying key opportunities for future research.

## 2. Meat and Dairy Fat Analogues: Products and Characteristics

Animal-sourced meat fats are typically rich in long-chain saturated and monounsaturated fatty acids; however, their distinctive physical and digestive properties arise not only from fatty acid composition but also from the molecular organization of triacylglycerols. The positional distribution of fatty acids on the glycerol backbone, together with their storage within adipocyte lipid droplets, governs fat crystallization, melting behaviour, and lipase accessibility during gastrointestinal digestion. Dairy fats, such as milk fats are highly complex, comprising over 400 fatty acids, and are naturally emulsified in globules surrounded by the milk fat globule membrane (MFGM). This structure plays a protective role during digestion and influences lipase access and hydrolysis kinetics [16]. These inherent complexities make replicating animal fats difficult. Analogues must therefore not only mimic composition but also account for structural factors that regulate digestion and nutrient release. This section summarizes the key plant- and microbial-based fat products that are currently available and their lipid profiles. Table 1 articulates the various types of meat products available, their structural lipid characteristics, and their nutritional implications.

**Table 1:** Comparative overview of lipid sources, structural characteristics, digestion behaviour, and nutritional implications in animal-, plant-, and microbial-derived fat systems.

Lipid Source/System	Structural Characteristics	Digestion Behaviour	Nutritional Implications	Reference
Animal-derived fats	Complex TAG positional distribution; adipocyte lipid droplets; milk fat globule membrane (MFGM)	Efficient lipase adsorption; rapid micelle formation	Promotes efficient bioaccessibility of fat-soluble nutrients	[17]
Plant oils (sunflower/canola)	PUFA-rich liquid TAGs; low solid fat fraction	Matrix-dependent lipolysis; altered hydrolysis kinetics	Bioaccessibility influenced by matrix viscosity and interfacial stability	[18]
Structured plant fats (coconut/palm blends)	Increased solid fat content; fat crystal networks	Slower melting and hydrolysis governed by crystallization behaviour	Modified fatty acid release kinetics due to solid fat structure	[19]
Plant-based dairy emulsions	Oil-in-water emulsions; homogenized lipid droplets	Droplet size and interface influence micelle formation	Affects delivery efficiency of fortified lipophilic nutrients (e.g., vitamin D)	[20]
Microbial oils (algae/yeast)	Intracellular lipid bodies; LC-PUFA-rich TAGs	Modified enzyme adsorption and lipid hydrolysis pathways	Alters lipid bioaccessibility and fatty acid release kinetics, influencing delivery of lipophilic nutrients	[21]

### 2.1. Plant-Derived Fat Analogues

Plant oils are the predominant fat substitutes in meat and dairy analogues owing to their availability, cost efficiency, scalability, and functional versatility. Analyses of commercial formulations and ingredient disclosures indicate that sunflower, canola, coconut, and palm oils are among the most frequently used lipid sources in analogue products [6,22], as they enable partial replication of animal fat properties such as melting behaviour, firmness, and oxidative stability.

Other vegetable oils, including olive, soybean, and corn oils, are also utilized, but to a lesser extent. Their limited use is largely attributed to pronounced flavour profiles (e.g., olive oil), increased oxidative susceptibility, or insufficient solid fat content at ambient temperatures, which restricts their functional equivalence to animal fats without additional structuring [6,23].

Nevertheless, commonly used plant oils differ markedly from animal fats in their fatty acid composition, particularly in terms of:

- High polyunsaturated fatty acids (PUFAs): Sunflower oil is characterized by high linoleic acid contents (≈70%), while canola oil is high in oleic acid and contains 7–12% α-linolenic acid. While this composition enhances nutritional value, it also increases susceptibility to oxidative degradation [24].
- Saturated fatty acids: Coconut and palm oils are rich in medium- and long-chain saturated fatty acids, which contribute to improved firmness and thermal stability. From a nutritional perspective, high intake of saturated fatty acids has traditionally been associated with increased cardiovascular risk according to dietary guidelines, although this relationship remains an area of ongoing scientific discussion [25].
- Structural modification strategies: Functional performance is often enhanced through interesterification, oleogelation, or emulsification, which alter melting characteristics, droplet size distribution, and lipase accessibility, thereby influencing lipid digestion and nutrient bioaccessibility [19].

#### 2.1.1. Plant-Derived Meat Products

The market for plant-based meat is expanding with increased social demand, with increasing emphasis on sensory quality and functionality. This category represents the largest segment of the meat analogue market and is projected to exceed US$21.23 billion by 2025 [26]. Plant-based meat analogues are prepared using plant-derived proteins such as wheat gluten, soybean, oilseeds, fungi, and legumes, which provide the primary structural matrix [27]. Among these, wheat gluten and soybean proteins are particularly prevalent due to their nutritional value and techno-functional properties, contributing approximately 8–17.5% and 35–40% protein, respectively. These proteins play a central role in matrix structuring and lipid emulsification, thereby enabling the effective incorporation and stabilization of added fats in plant-based meat systems [28,29].

To compensate for the absence of animal adipose tissue, plant-based meats are supplemented with vegetable oils (e.g., coconut oil and cocoa butter for mimicking the melting behaviour, juiciness, and mouthfeel of animal fat) [26]. Consequently, many products exhibit higher total fat and energy values compared with conventional meat. While lipid addition is essential for sensory realism, it also influences the overall nutritional profile, including saturated fat levels. Despite the continuous improvements, achieving desirable palatability remains a significant challenge. Texture and flavour development depend not only on protein structuring techniques (e.g., spinning, thermoplastic extrusion, and steam texturization), but also on the effective distribution and stabilization of lipids within the matrix [30]. Another challenge in plant-based meats is their appearance (e.g., colour), which further influences consumer acceptance. To achieve the red colour similar to traditional meat, beetroot extract or tomato paste is added in some plant-based meat products [26], but it does not always appear red due to changes in chemical state of myoglobin, which is primarily responsible for meat colour. For this, leghemoglobin is used in meat analogues, which have chemical structure similar to myoglobin (e.g., Impossible Burger by Impossible Foods Inc., Redwood City, CA, USA) [31].

#### 2.1.2. Plant-Derived Dairy Products

The rapid growth of plant-based dairy products, such as milk, cheese, yoghurt, cream and butter alternatives has been driven by consumer demand for lactose-free, allergen-free, and sustainable options. These products are typically formulated from plant proteins (soy, almond, oat, coconut, pea) combined with plant oils to replicate the texture, mouthfeel, and nutritional properties of cow’s milk and dairy fats [32,33]. The global market for plant-based dairy products is expanding rapidly, projected to reach USD 34 billion by 2030, with plant-based milks accounting for approximately 50% of total sales. The Asia–Pacific region currently leads in consumption volume, reflecting a strong demand for lactose-free and sustainable alternatives [34].

Plant-based milk is typically formulated as oil-in-water emulsions, where dispersed plant oils provide creaminess and energy density. The stability and digestive behaviour of these systems depend on droplet size, homogenization, and interfacial stabilization by proteins or emulsifiers, which also influence the bioaccessibility of fortified fat-soluble nutrients such as vitamin D [18,20].

In plant-based cheeses, lipid systems (such as coconut oil or structured vegetable oil blends) govern firmness and meltability. Unlike dairy milk fat, which exhibits complex triacylglycerol crystallization behaviour, plant oils often require structuring through blending, emulsification, or starch incorporation to achieve appropriate thermal and textural properties [35,36]. Similarly, in plant-based yoghurts and spreads, lipid droplets interact with protein–polysaccharide networks, affecting mouthfeel, phase stability, solid fat content, and fat crystal polymorphism, all of which present ongoing formulation challenges [37].

### 2.2. Microbial-Derived Fat Analogues

Recent advances in biotechnology have enabled microbial production of lipids through precision fermentation. Microbial fats can be engineered to resemble animal fats more closely in terms of fatty acid composition and melting profiles. For example, yeast and microalgae can be tailored to produce higher levels of saturated or monounsaturated fatty acids [21], with certain strains capable of synthesizing bioactive lipids such as omega-3 fatty acids [38]. This approach provides a modifiable approach to synthesizing lipids with specific chemistries and compositions to tailor the fat profile of meat analogues.

Microbial fermentation platforms also contribute to fat substitution through lipid-rich biomass and fermentation-derived oils with tailored structural properties. Oleaginous fungi, yeasts, and microalgae can accumulate intracellular lipids that influence lubrication, mouthfeel, and flavour release, supporting the functional replacement of animal adipose tissue in meat and dairy analogues [39,40]. In particular, microalgal systems such as *Schizochytrium* enable the production of long-chain polyunsaturated fatty acids that modify emulsion stability and nutritional value [41]. Emerging precision fermentation strategies are increasingly directed toward generating structured lipid phases with defined fatty acid composition and melting behaviour, offering potential to replicate the crystallization characteristics and functional performance of conventional meat and dairy fats while maintaining sustainable production pathways [42,43].

## 3. The Impact of Lipid Structure and Composition on Digestion in Fat Analogues

In meat and dairy analogues, lipid digestion is governed not only by fatty acid composition but, more critically, by the structural organization of lipids within the food matrix. Although total fat contents typically range from 0 to 15%, lipids in analogue products are incorporated into complex systems comprising proteins, carbohydrates, water, and stabilizing agents, which collectively regulate lipid accessibility and enzyme–substrate interactions during gastrointestinal digestion [44].

Key structural features, including lipid physical state (solid versus liquid), droplet size distribution, degree of emulsification, and interfacial composition strongly influences the lipolysis kinetics, mixed micelle formation, and subsequent nutrient solubilization. While plant-derived oils are generally richer in unsaturated fatty acids and are often considered nutritionally advantageous relative to animal fats, their distinct molecular and supramolecular organization results in digestion behaviour that differs substantially from animal-derived lipids [45].

**Figure 1:**
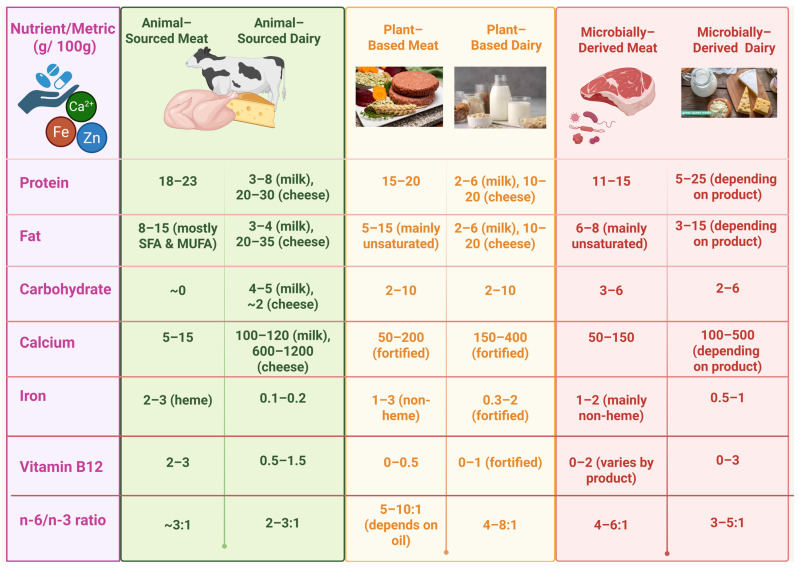
Comparison of animal-, plant-, and microbially sourced meat and dairy composition and nutritional value (g/100 g).

As a result, differences in lipid structuring strategies and matrix interactions, rather than lipid source alone, play a decisive role in shaping gastrointestinal fate, lipid digestion efficiency, and the bioaccessibility of co-ingested lipophilic nutrients in fat analogue systems [46]. A nutritional comparison in the properties of animal-, plant-, and microbial-derived meat is provided in Figure 1.

### 3.1. Effect of Lipid Emulsion Structure

**Figure 2:**
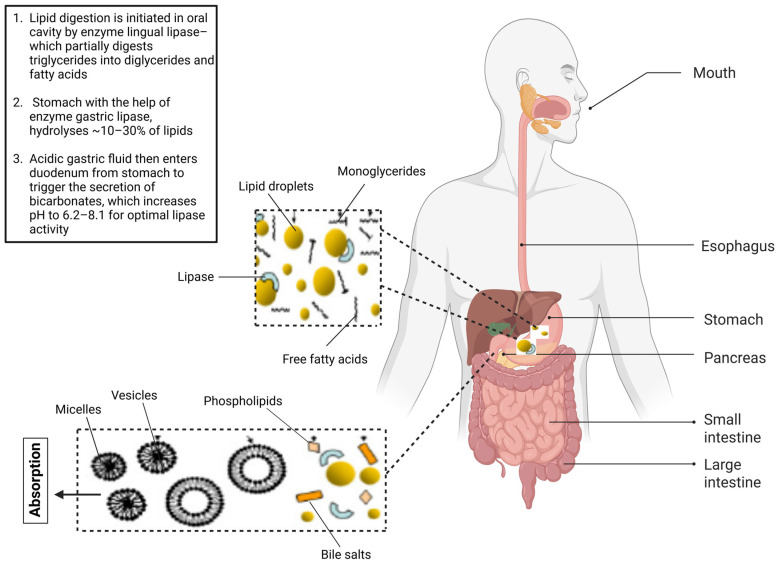
Schematic representation of the digestive process for endogenous lipids. Lipid droplets are hydrolysed by lipases to form monoglycerides and free fatty acids, which interact with bile salts and phospholipids to form mixed micelles prior to absorption in the small intestine. Arrows indicate the direction of lipid digestion and transport, while schematic icons represent lipid droplets (yellow spheres), phospholipids (curved structures), bile salts (orange rods), and mixed micelles (circular vesicles).

Fat digestion begins with emulsification and continues through enzymatic hydrolysis by gastric and pancreatic lipases (Figure 2). The rate of lipolysis depends strongly on droplet size, interfacial composition, and supramolecular structure, given that lipase is an interfacially active enzyme that relies on access to the lipid-in-water interface to digest triglycerides into fatty acids and monoglycerides [47,48,49]. For example, dairy fats are naturally emulsified within milk fat globules, a complex tri-layer of phospholipids, glycoproteins, and bioactive compounds that influences lipase access and digestion kinetics [50]. In contrast, most plant-based dairy alternatives rely on homogenized emulsions stabilized by proteins or hydrocolloids, which rarely mimic the MFGM structure and alter the bioaccessibility of the lipid-in-water interface to the lipase enzyme. Studies using in vitro digestion models frequently report slower lipase binding and delayed micelle formation in plant-based emulsions compared with dairy systems; however, variability in digestion protocols and interfacial design limits the direct comparability between studies [51,52]. Similarly, in meat analogues, fat droplets are either added as oils dispersed in protein matrices or structured into oleogels, which alters lipase accessibility compared with intramuscular fat in animal meat [53,54].

### 3.2. Effect of Fatty Acid Chain Length

The chain length of fatty acids plays a central role in their digestibility and absorption. Medium-chain fatty acids (MCFAs), abundant in coconut oil used in many analogue formulations, are hydrolysed rapidly, absorbed directly into the portal vein, and metabolized efficiently for energy. By contrast, long-chain fatty acids (LCFAs), particularly saturated ones, are digested more slowly, require re-esterification, and are transported via the lymphatic system [55]. This is due to the inherent hydrophobicity of LCFAs and their tendency to partition and adsorb at the emulsion interface, and thus, impede lipid digestion to a far greater degree than for MCFAs [56].

Microbial oils are notable for their ability to produce customisable fatty acid profiles, including long-chain polyunsaturated fatty acids (LC-PUFAs) such as EPA and DHA, which are typically absent in plant-based oils. While these fatty acids provide nutritional advantages, their digestion and micellization efficiency depend on chain length and degree of unsaturation. LC-PUFAs are more slowly hydrolysed than medium-chain fatty acids (MCFAs) and require efficient emulsification for intestinal absorption. Precision-fermented lipids designed to mimic the triacylglycerol (TAG) profiles of dairy fat may also influence digestion kinetics by reproducing positional distribution patterns (sn-2 palmitic acid), which are known to enhance lipid absorption and calcium uptake in infants [57,58].

Emerging evidence positions precision-fermented fats as a distinct class of engineered lipids, enabling the biosynthesis of structured triacylglycerols with controlled fatty acid composition and positional distribution. Unlike conventional plant oils, these lipids can be engineered to reproduce structural features of animal fats, such as sn-2 palmitic acid enrichment or tailored melting profiles, which may influence enzyme accessibility, lipolysis kinetics, and mixed micelle formation during digestion. Although early studies suggest that precision-fermented lipids could restore digestion behaviour closer to dairy-derived fats, current evidence remains limited and largely based on compositional modelling or in vitro simulations. Consequently, systematic comparisons using harmonized digestion models are required to determine whether these engineered lipids consistently improve nutrient bioaccessibility relative to conventional plant-based fat systems [57,58].

### 3.3. Effect of Food Matrix Composition

The digestion of analogues depends not only on fat type but also on the matrix composition, including proteins, carbohydrates, and fibre, unlike animal products, which mainly consist of protein and fat, plant-based analogues contain significant levels of digestible and indigestible carbohydrates, such as starch and dietary fibre. These components can modulate digestion by altering viscosity, emulsification stability, and nutrient release patterns [59]. That is why, it is important to understand the health and nutritional impacts due to switching from omnivore to a vegetarian/vegan diet [60].

Meat analogues are designed to mimic the taste and nutritional functionality of animal meat. Generally, they contain high amount of protein with essential amino acids and low-fat content which make them beneficial for human health [6]. While dietary fibre is added to improve the textural properties and water holding capacity of meat analogues [61]. Despite these nutritional characteristics, the behaviour of these nutrients in gastrointestinal tract is unclear [62]. Plant-based meat analogues have different composition and structural properties as compared to animal meat products, which explains the reason behind the difference in gastrointestinal fate of meat analogues. Also, the addition of additives, colouring agents and flavours changes the digestion behaviour, absorption, and integration of nutrients in meat analogues [63].

Zhou et al. (2021) [64] studied the animal meat and meat analogues properties to compare their gastrointestinal fate by using the in vitro (INFO-GEST) digestion model, while the focus was on physicochemical and microstructural properties. The results of this study concluded that the presence of dietary fibre in plant-based meats slowed the lipid digestion in the small intestine, which resulted in an increase in lipid viscosity in gastric and intestinal fluids. Similarly, it is found from other studies that addition of various ingredients in meat analogue formulations hinder or delay the digestion; therefore, it is recommended to exclude the ingredients which cause obstruction in the digestion process [65].

Wilde et al. [66] found in their study that gastrointestinal fate and digestion of lipids in the gastrointestinal tract depends on their biological source, e.g., lipids obtained from oats are resistant to digestion, due to their tendency to aggregate under gastrointestinal conditions, their interfacial coating’s resistance to enzyme hydrolysis, and the presence of phytosterols that interfere with the digestion process. However, proteins and lipids extracted from almonds are more digestible under simulated GIT conditions, as they aggregate under gastric conditions and their endogenous proteins and peptides remain at their surfaces in the stomach. After passing into the small intestine, these lipids become less aggregated due to the increase in pH and bile salts presence and are ultimately digested by proteases, phospholipases, and lipases [67].

Furthermore, anti-nutritional factors such as phytochemicals (tannins, phytates, lectins and trypsin inhibitors) limits the digestibility of plant proteins. To overcome this problem, inactivation of these anti-nutrients is suggested by using several processing techniques such as cooking, microwave processing, extrusion, autoclaving, fermentation, freeze-drying, and irradiation [68]. However, impact of these processing techniques on plant proteins needs to be explored further. Also, there is a need for in depth study of simulated gastrointestinal models with regard to digestion fate of nutrients present in meat analogues. Similarly, in vitro and in vivo trials should be conducted to assess the difference in bioavailability and nutritional value of animal meat versus plant-based meat by studying their absorption rate in gastrointestinal tract.

Microbially sourced fats, in particular mycoprotein-based analogues, are typically low in intrinsic fat, with lipid profiles defined by the addition of plant oils such as rapeseed or sunflower. The rigid fungal cell wall, rich in chitin and β-glucans, can encapsulate lipids and proteins, reducing their accessibility to digestive enzymes [69]. In vitro studies suggest that this microstructural barrier slows lipolysis and delays the release of fatty acids compared with conventional meat. In contrast, lipids derived from algae or oleaginous yeasts are frequently incorporated as emulsified or structured lipid systems characterized by phospholipid-enriched interfaces and intracellular lipid body structures that differ from protein-stabilized plant emulsions, potentially altering lipase binding, interfacial displacement, and fatty acid release kinetics [70].

The composition of microbially derived analogues including proteins, polysaccharides, and secondary metabolites can further modulate lipid digestion. For instance, β-glucans in fungal biomass may increase gastric viscosity and delay lipid release, while phytochemicals in algae can interfere with bile salt activity and micelle formation [71]. Conversely, microbial dairy analogues produced via precision fermentation (e.g., animal-free milk fats and caseins) are being designed to closely reproduce the lipid–protein assemblies of bovine milk, which may restore efficient lipid digestion and nutrient delivery.

### 3.4. Effect of Processing Conditions and Lipid Oxidation

In addition to compositional differences, processing conditions such as emulsification, high-temperature, extrusion, homogenisation, and oleogel formation can significantly influence lipid structure and subsequent digestion behaviour. Each of these processes results in stable oil-in-water or water-in-oil emulsions, increasing the surface area of the lipid interface, irrespective of lipid type. As stated in Section 3.1, this increase in interfacial surface area facilitates and improves the bioaccessibility for the lipase enzyme to bind to and digest the triglyceride substrate, resulting in enhanced lipolysis kinetics [72,73]. However, each of these processes, together with storage conditions, can promote lipid oxidation, since increasing the oil–water interfacial area allows for pro-oxidants (e.g., iron) present in the aqueous phase to interact more readily with unsaturated fatty acids [74,75]. This is especially important for plant and microbial oils that are rich in polyunsaturated fatty acids, given their increased tendency for oxidation. Together with the utilization of advanced packaging (e.g., vacuum and modified atmosphere packaging), Natural antioxidants (e.g., rosemary extract, phenolic compounds, vitamin C and E) are typically added to plant- and microbial-based meats and dairy products [76], to inhibit free radical formation, but preventing lipid oxidation remains a challenge [77].

Ultimately, lipid oxidation has important ramifications for digestion efficiency, shelf-life, food safety, and downstream health outcomes. Oxidative modification inhibits lipase activity through a multitude of mechanisms [78]. Firstly, it compromises food structure and lowers emulsification capacity, leading to a reduced capacity for lipase to access the oil–water interface. Further, oxidized lipids can form complex compounds that are physicochemically resistant to lipase hydrolysis, while some secondary products of oxidation (e.g., malondialdehyde) can denature digestive enzymes [79]. This highlights that further research is critically required to clarify how processing-induced lipid oxidation influences gastrointestinal behaviour in analogue products.

## 4. Implications of Variable Lipid Digestion Behaviour in Meat and Dairy Fat Analogues

The composition and structure of lipids in meat analogues strongly influence their digestive behaviour and subsequent bioavailability of lipophilic nutrients and vitamins. Plant-based meat alternatives generally contain lower levels of saturated fatty acids and cholesterol, but higher proportions of unsaturated fats and dietary fibre compared with animal meat [26]. These differences impact overall fat and protein digestibility: while plant-based proteins may exhibit faster gastric protein breakdown, their lipid hydrolysis and nutrient absorption are often lower than in animal meat [64,80]. Microbial-derived meat analogues, such as mycoprotein from Fusarium venenatum, provide a more complete amino acid profile than most plant sources and moderate digestibility [81]. However, fibrous cell wall structures can slow lipid release, affecting micelle formation and the bioavailability of fat-soluble vitamins and minerals.

Altered lipid digestion in meat and dairy analogues affects micronutrient absorption, energy availability, and satiety, with broader health implications, including:

- Micronutrient bioavailability: Reduced lipid digestion can limit the absorption of fat-soluble vitamins and minerals. Plant-based meats are particularly susceptible to low bioavailability unless fortified, while microbial analogues may help bridge some gaps [82].
- Satiety and metabolic regulation: Slower lipid hydrolysis can influence hormonal responses related to hunger and fullness, influencing overall energy intake [83].
- Long-term health outcomes: Differences in fatty acid composition (e.g., high polyunsaturated fats in plant or microbial-derived systems) may contribute to cardiovascular benefits, while insufficient micronutrient uptake could increase deficiency risks [84].

As discussed mechanistically in Section 3, differences in lipid organization, interfacial composition, and food matrix interactions collectively govern lipolysis kinetics and mixed micelle formation. Here, these structural factors are considered primarily in terms of their nutritional implications rather than digestion mechanisms. In plant-based systems, factors such as reduced saturated fat content, altered fatty acid chain length, and the presence of anti-nutritional compounds (e.g., phytates and polyphenols) may reduce bioavailability compared with animal products. Conversely, fortification strategies, emulsification technologies, and advanced delivery systems (e.g., nano-encapsulation) are increasingly being applied to mitigate these challenges and enhance nutrient delivery. Beyond nutritional outcomes, altered lipid bioaccessibility in analogue systems may also have implications for regulatory assessment and nutritional labelling. Existing frameworks are largely based on compositional nutrient content rather than functional bioavailability; however, engineered lipid structures may influence the effective delivery of lipophilic nutrients during digestion. As precision-fermented and structurally modified fats continue to emerge, future regulatory guidance may need to consider how lipid architecture and gastrointestinal behaviour affect nutrient claims and functional equivalence with animal-derived products.

In addition to regulatory considerations, these mechanistic insights also carry practical implications for food formulation. These findings highlight the importance of lipid structuring strategies beyond simple fatty acid substitution. Control of droplet size, interfacial composition, and solid–liquid fat balance play an important role in optimizing lipase accessibility, lipolysis kinetics, and nutrient bioaccessibility in analogue systems. For example, structured emulsions, oleogels, and precision-fermented lipids offer opportunities to design fat systems that more closely replicate the digestive behaviour of animal-derived fats while maintaining desired sensory and sustainability attributes. Consequently, future product development may increase focus on tailoring lipid architecture at the microstructural level to balance technological performance with nutritional functionality.

Despite substantial progress in understanding lipid digestion mechanisms, several limitations should be acknowledged. Much of the current evidence is derived from static or semi-dynamic in vitro digestion models, which differ considerably in enzyme activity, shear conditions, and bile composition, making cross-study comparisons challenging. Furthermore, while these models provide valuable mechanistic insight, their ability to predict long-term nutritional outcomes or health effects remains limited. There is currently a lack of longitudinal human studies examining lipid digestion and nutrient bioavailability in meat and dairy analogue systems, and therefore conclusions regarding health impacts should be interpreted with caution.

Collectively, these insights demonstrate that variability in lipid digestion is not merely a compositional issue but a structural design challenge that links food formulation with nutritional functionality.

## 5. Challenges and Current Knowledge Gaps for the Digestion of Fat Analogues

Despite growing interest in analogue fat systems, several research gaps remain, largely due to the over-reliance on in vitro lipolysis models to simulate the gastrointestinal digestive environment. While in vitro digestion models provide valuable mechanistic insight into lipid hydrolysis and micelle formation, important differences exist between static simulations, animal studies, and human digestion. In vitro systems often simplify gastric shear, enzyme secretion dynamics, and bile variability, which may lead to over- or under-estimation of lipolysis kinetics compared with in vivo conditions [85]. Animal models offer greater physiological complexity but differ in gastrointestinal transit time and lipid metabolism relative to humans. Standardized digestion protocols are lacking, making cross-study comparisons difficult, and long-term human trials evaluating lipid digestion and nutrient bioavailability in analogue products are still scarce. Furthermore, interactions between processing-induced structural changes and lipid digestion remain insufficiently understood, highlighting the need for multidisciplinary approaches combining food engineering, nutrition science, and clinical evaluation.

Consequently, findings derived from model systems should be interpreted cautiously, and future research should prioritize integrated human studies to validate digestion behaviour and nutritional outcomes in analogue fat systems. This knowledge will aid in further understanding how changes in lipid digestion profiles impact human nutritional outcomes, with clinical studies also providing an avenue to understand the use of fat analogues within a broader dietary context, the impact of individual metabolic variability, and long-term consumption patterns.

## 6. Conclusions and Future Directions

Rising concerns around sustainability, health, and ethics are accelerating the development of plant-based food products and microbial-derived food systems. Compared with emerging alternatives, such as cultured meat, plant-based products currently benefit from a more established supply chain, lower production costs, and faster regulatory approval, making them commercially viable in the short term. Nevertheless, cultured and precision-fermented foods continue to face technical, economic, and regulatory barriers, including scaling challenges, bioprocess optimization, and evolving safety frameworks.

Microbial fermentation, including mycoprotein-based and precision-fermented meat analogues is emerging as a particularly promising approach. Fungi, yeast, and bacteria can be used to produce high-protein biomass with meat-like textures, offering advantages such as high production efficiency, minimal land use, and reduced greenhouse gas emissions. Advances in synthetic biology, metabolic engineering, and fermentation technology are likely to further improve the nutritional profile, texture, and functional properties of these products. Future research should focus on several critical areas:

- Optimizing digestibility and nutrient bioavailability: Understanding lipid and protein digestion in microbial and plant-based matrices is essential to ensure analogues provide comparable or superior nutritional value to animal products. Innovations in lipid structuring, protein engineering, and microencapsulation can enhance nutrient delivery.
- Texture and sensory improvements: Advanced processing technologies, such as extrusion, 3D food printing, and fermentation-mediated texturization, are needed to mimic the sensory experience of traditional meat and dairy products.
- Sustainability and life cycle assessment: Comprehensive evaluation of environmental impacts, including water use, energy consumption, and carbon footprint, will guide more sustainable production practices.
- Regulatory and safety frameworks: Harmonizing global regulations for novel proteins, particularly precision-fermented and cultured products, is crucial for widespread adoption. Safety, allergenicity, and labelling standards require ongoing assessment.
- Consumer acceptance and market integration: Social, cultural, and economic factors influence adoption. Research into consumer perceptions, pricing strategies, and education campaigns will help increase acceptance of microbial and cultured products.

Rather than being driven solely by technological convergence, the advancement of meat and dairy fat analogues will depend on a mechanistic understanding of how lipid structure, interfacial design, and food matrix interactions govern digestion and nutrient bioaccessibility. The framework presented in this review links lipid architecture to gastrointestinal behaviour, guiding the rational design of next-generation fat analogues with controlled functionality and improved nutritional performance. Importantly, these findings highlight that nutritional equivalence between animal-derived and analogue products cannot be assumed solely based on compositional similarity. Instead, lipid digestion must be evaluated alongside protein digestion to provide a more accurate assessment of nutrient delivery. From a formulation perspective, future analogue design will likely rely on structure-driven lipid strategies that integrate food engineering with digestion-focused nutritional assessment.

## Data Availability

No new data were created or analyzed in this study. Data sharing is not applicable to this article.
